# RGTA^®^ or ReGeneraTing Agents mimic heparan sulfate in regenerative medicine: from concept to curing patients

**DOI:** 10.1007/s10719-016-9744-5

**Published:** 2016-12-07

**Authors:** Denis Barritault, Marie Gilbert-Sirieix, Kim Lee Rice, Fernando Siñeriz, Dulce Papy-Garcia, Christophe Baudouin, Pascal Desgranges, Gilbert Zakine, Jean-Louis Saffar, Johan van Neck

**Affiliations:** 1 OTR3, 4 rue Française, 75001 Paris, France; 2 Laboratory Cell Growth and Tissue Repair (CRRET), UPEC 4397/ERL CNRS 9215, Université Paris Est Cretéil, Université Paris Est, F-94000 Créteil, France; 3 Institut de la Vision, 17 rue Moreau, 75012 Paris, France; 4 Universite Paris-Saclay, Université de Versailles Saint-Quentin-en-Yvelines (UVSQ), 55 Avenue de Paris, 78000 Versailles, France; 5 Centre Hospitalier National d’Opthalmologie des Quinze Vingts, 28 rue de Charenton, 75012 Paris, France; 6 Department of Vascular Surgery, Hopital Henri Mondor, Université Paris-Est Créteil, 51 avenue du Maréchal de Lattre de Tassigny, 94000 Créteil, France; 7 Service de Chirurgie Plastique et Reconstructrice, 33 rue de la Tour, Paris, 75016 France; 8 EA2496 Laboratoire Pathologies, Imagerie et Biothérapies Oro-Faciales, Faculté de Chirurgie Dentaire, Université Paris Descartes, Sorbonne Paris Cité, 1 rue Maurice Arnoux, 92120 Montrouge, France; 9 Department of Plastic and Reconstructive Surgery, Erasmus MC - University Medical Center, Rotterdam, The Netherlands

**Keywords:** RGTA^®^, Heparan sulfate mimics, Extracellular scaffold, Regeneration

## Abstract

The importance of extracellular matrix (ECM) integrity in maintaining normal tissue function is highlighted by numerous pathologies and situations of acute and chronic injury associated with dysregulation or destruction of ECM components. Heparan sulfate (HS) is a key component of the ECM, where it fulfils important functions associated with tissue homeostasis. Its degradation following tissue injury disrupts this delicate equilibrium and may impair the wound healing process. ReGeneraTing Agents (RGTA^®^s) are polysaccharides specifically designed to replace degraded HS in injured tissues. The unique properties of RGTA^®^ (resistance to degradation, binding and protection of ECM structural and signaling proteins, like HS) permit the reconstruction of the ECM, restoring both structural and biochemical functions to this essential substrate, and facilitating the processes of tissue repair and regeneration. Here, we review 25 years of research surrounding this HS mimic, supporting the mode of action, pre-clinical studies and therapeutic efficacy of RGTA^®^ in the clinic, and discuss the potential of RGTA^®^ in new branches of regenerative medicine.

## Introduction

The processes governing tissue repair and regeneration involve the interplay between cells and signaling molecules, within the context of a three dimensional extracellular matrix (ECM). A growing number of observations have demonstrated that heparan sulfates (HS) are essential components of the ECM, *via* their ability to store, protect and regulate the bioavailability of communication factors required for normal one-to-one cell replacement or tissue repair [1]. The destruction of HS following tissue injury, liberates cytokines from this protective matrix compartment and destabilizes the tissue microenvironment.

RGTA^®^s (ReGeneraTing Agents) are chemically engineered polymers that are specifically designed to replace degraded HS in the injured matrix compartment. By sequestering proteins through low affinity binding, RGTA^®^ protects naturally existing structural and signaling proteins, and in doing so, enhances the speed and quality of tissue repair. The mechanism of action and effects of RGTA^®^ have been investigated in many systems of tissue injury, with often remarkable results. In this review, we summarize our current understanding of the mode of action of RGTA^®^ and provide key examples of RGTA^®^’s efficacy in pre-clinical models and in humans. These studies demonstrate that RGTA^®^-based restoration of the ECM and triggering of tissue regeneration represent an effective way to treat diverse pathologies, constituting a clinically evidenced, new branch of regenerative medicine.

## The extracellular matrix and its role in tissue homeostasis

The ECM is a network of macromolecules that includes a plethora of structural proteins, enzymes and soluble factors, which interact in a dynamic fashion with surrounding cells, in order to maintain tissue structure and homeostasis. The ECM can be divided into the interstitial connective tissue matrix, a structural tissue component surrounding cells and the basement membrane, which delineates the epithelium from the underlying tissue. Analysis of the mammalian ‘matrisome’ has defined a list of approximately 300 central ECM proteins. These include **collagens**, the major structural components of the ECM, **proteoglycans** (**PGs**), core proteins with attached glycosaminoglycan (GAG) chains that sequester water and bind growth factors, and **glycoproteins**, which play diverse roles in ECM structure and communication, and also act as a reservoir for growth factors. In addition to these central proteins, the ECM also includes numerous other proteins such as enzymes, growth factors and cytokines, which play important roles in homeostasis and remodeling [2]. The ECM of each tissue is a unique composition of these elements in three dimensional space and controlling the delicate balance of enzymatic and non-enzymatic activities is crucial for normal tissue function. This is underscored by the existence of numerous pathologies associated with dysregulated ECM components including osteoarthritis, fibrosis and cancer [3, 4].

HSs are a subclass of sulfated GAGs that play a key role in the regulation of tissue homeostasis by mediating and integrating communication events. In the ECM, these exist as heparan sulfate proteoglycans (HSPGs), which comprise a core protein to which one or several HS chains are covalently linked. HSPGs may be expressed on the surface of cells (syndecans, glypicans), secreted in the ECM (agrin, perlecan, type XVIII collagen) or in secretory vesicles (serglycin). In brief, HS is a crucial element of the ECM, fulfilling both an:

1. *Architectural Role*: HSPGs bind matrix proteins such as collagens, fibronectin and laminin at specific ‘heparan sulfate binding sites’, protecting these proteins from proteolytic degradation and forming a scaffold around cells, thereby contributing to the spatial organization of the ECM [1] and a
2. *Protection Role*: The structural complexity (degree of substitution and position of sulfate groups) of HS is diverse, and allows HS to specifically bind peptides and ECM matrix proteins [5]. By binding growth factors, cytokines and chemokines, HSPGs protect factors from degradation and control their retention, thereby regulating their local concentration and signaling capabilities. In some cases, factors remain bound to HS upon binding their respective receptors, such that the HSPG ‘presents’ the ligand to its respective receptor, whereas in other cases cleavage of the factor from HS is required for function [1].

Extensive tissue damage is typically followed by normal wound repair, a sequence of events involving hemostasis and inflammation, proliferation and tissue remodeling. During this process, cells involved in the inflammatory response (macrophages, neutrophils) and resident cells at the wound site (keratinocytes, fibroblasts and endothelial cells) release heparanases and proteases into the injured tissue [6]. This leads to the degradation of HS, destruction of the ECM scaffold, and the release of HS-bound communication polypeptides into the ECM, stimulating the rapid repair of tissue, and is accompanied by resolution of the inflammatory response. This repair process results in scars or fibroses, and while the resultant tissue provides a barrier to prevent further infection, the quality of tissue is lower, both structurally and aesthetically. In the case of chronic wounds, however, such as those associated with diabetes mellitus and vasculitis, the wound remains in a state of chronic inflammation. The increased proteolytic activity, caused by persisting inflammatory cells, leads to degradation of ECM, structural proteins and growth factors required for repair, and the ECM microenvironment is trapped in a cycle of repair and destruction [6].

## RGTA^®^ matrix technology – Heparan sulfate mimics in tissue regeneration

Matrix therapy is based on the concept of restoring the cellular microenvironment, which is disrupted following tissue injury, with the aim of promoting communication between cells and re-establishing tissue homeostasis. ReGeneraTing Agents are heparan sulfate mimics, specifically designed to replace degraded heparan sulfates in damaged tissue, accelerating the speed and enhancing the quality of tissue repair. Their unique properties have been subject of intensive pre-clinical studies and we are now at the point where the clinical utility of these polymers is being fully realized.

### RGTA^®^ – Chemical structures and stability

RGTA^®^s are chemically substituted polymers with sulfates and carboxyl groups, to which other groups such as fatty acids, amino acids or other substances of pharmacological interest may be added in specific amounts and locations, based on their performance *in vitro* (interaction with matrix proteins and growth factors) and *in vivo* (tissue regeneration capabilities). Importantly, this hemisynthetic pathway is highly controlled, allowing the design and production of well characterized molecules with significant therapeutic value [7]. While several other substituted polysaccharides or copolylactic and malic copolymer backbones also fulfil the RGTA^®^ definition [8], dextran has been preferred for reasons of safety and accessibility.

Although structurally and functionally analogous to naturally derived HS, one of the most crucial properties of RGTA^®^ is its resistance to enzymatic degradation. This allows RGTA^®^s to retain their structure and activity even in the microenvironment of chronic wounds, characterized by unrestrained proteolytic activity [6, 9]. In injured tissue, HS, present at the cell surface and within the ECM, is degraded by heparanase, and other ECM components, required for wound healing and homeostasis, are destroyed by locally secreted proteases. Unlike HS disaccharide subunits, which are linked by β1-4 carbon-carbon bonds sensitive to enzymatic cleavage, RGTA^®^ subunits are linked by α1-6 carbon-carbon bonds, rendering RGTA^®^ completely resistant to degradation by endoglycosidases known to digest natural GAGs, including chondroitinase ABC, dextranase, hyaluronidase and a mixture of heparitinases I, II and III [9]. In this way, RGTA^®^ can replace destroyed HS, reversing the hostile microenvironment and fostering tissue healing.

### RGTA^®^ mode of action - Structural and protective roles

**Fig. 1:**
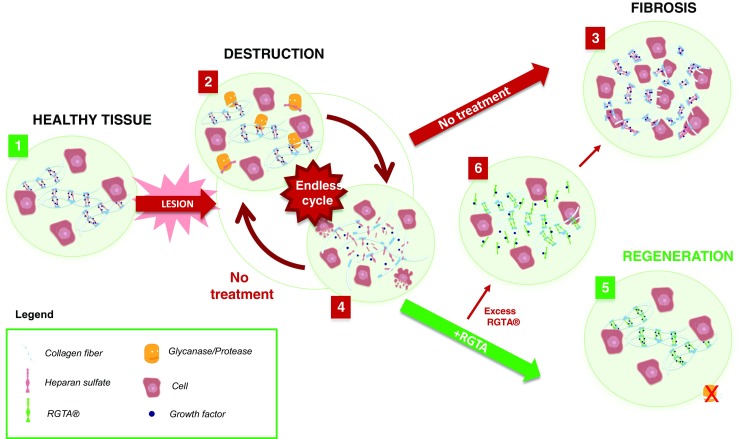
Mode of Action of ReGeneraTing Agent (RGTA^®^) Following Extensive Tissue Damage. 1. The Extracellular Matrix (ECM) organization in healthy tissue (left panel). Blue dots correspond to communication peptides, stored and protected by glycosaminoglycans. Heparan sulfate (HS) (light pink helices) links the matrix proteins (white fibers) to form a scaffold around cells. 2. Following extensive tissue damage, HS is degraded by glycanase (yellow figurines), the ECM scaffold structure is destroyed and proteases (yellow figurines) may enter, destroying matrix proteins and stored growth factors. 3. A rapid repair response is triggered resulting in scars or fibroses. 4. If the cause of the lesion is not resolved, wounds remain in an inflammatory phase, leading to a cycle of repair and destruction. 5. RGTA^®^ (green helices) replaces degraded HS, allowing reconstruction of the ECM scaffold and restoring the cellular microenvironment. This facilitates tissue regeneration and restoration of tissue to a state similar to that of healthy tissue. 6. Excess RGTA^®^ may hinder the healing process by drawing growth factors and communication peptides away from the wound site. In this situation, the bioavailability of growth factors required for efficient wound healing is reduced, emphasizing the need to optimize the RGTA^®^ treatment protocol (dose and timing) in accordance with the tissue specific matrix turnover

The degradation of HS at the site of injured tissue, leads to the disorganization of the ECM and exposes structural and communication-proteins to protease activity. RGTA^®^ fosters restoration of the natural architecture of the ECM by specifically localizing to the injured tissue [10], where it may bind free ‘heparan binding sites’ present in structural proteins such as collagen, fibronectin and laminin [11]. This binding has two important consequences. Firstly, it serves to protect the matrix proteins from proteolytic degradation and secondly, it facilitates reconstruction of the ECM scaffold, a necessary first step in reestablishing a microenvironment conducive to tissue repair (Fig. 1).

The ability of RGTA^®^ to protect and thereby potentiate signaling peptides and growth factors, re-establishing the ECM communication network, is another key feature underlying the therapeutic action of RGTA^®^ and is well documented. In chronic wounds, the levels of growth factors required for matrix formation, remodeling, formulation of granulation tissue and re-epithelialization are decreased. This is due, in part, to the high levels of proteolytic enzymes released by inflammatory cells as well as the presence of reactive oxygen species. RGTA^®^ binds numerous heparin-binding growth factors including FGF [12], VEGF [13] and TGFβ [14], or chemokines such as SDF-1 [15] and in doing so, controls their bioavailability, protects them from proteolytic degradation [16–18] (which may be due to the protective effect of direct binding and/or due to the ability of RGTA^®^ to inihibit enzymes such as plasmin or neutrophil elastase) and may also increase affinity for their receptors [12, 13]. By reestablishing the spatio-temporal distribution of growth factors, RGTA^®^ may influence important processes contributing to tissue healing and regeneration such as cell migration and differentiation [19, 20] and angiogenesis [13], in addition to providing general protection against oxidative stress [21] and apoptosis [22].

Our current understanding of RGTA^®^’s mode of action points to a relatively simple mechanistic model, whereby RGTA^®^ readily replaces degraded GAGs and particularly HS, in an altered ECM. In this way, RGTA^®^ selectively protects heparin binding cytokines and structural proteins, facilitating natural matrix reorganization and favoring tissue homeostasis and/or repair. Although no specific receptors for RGTA^®^s are known, one can consider that because of its capacity to interact with a diversity of HS binding proteins in the ECM, RGTA^®^ is a HBP low affinity receptor mimic. Moreover, since RGTA^®^ is biodegraded when internalized and catabolized through lysosomal pathways within cells (Barritault, unpublished data), as for other matrix elements, other possible roles for RGTA^®^ at the intracellular level can be discounted. However, to strongly support this hypothesis, further studies are required and this is the focus of ongoing research. The turnover of the matrix constituents is tissue specific, and is also dependent on the degree of the injury. The extent of RGTA^®^ induced restoration will depend on the dose and frequency of application. Once reorganization of the matrix is achieved, and matrix protein binding sites are saturated, additional RGTA^®^ may in fact be deleterious, with excess, unbound RGTA^®^ attracting already bound factors away from the site of injury (Fig. 1). Hence dosing and timing need to be adaped to reflect the matrix turnover, which varies from tissue to tissue and according to the stage of healing. By restoring the extra-cellular microenvironment, RGTA^®^ favors the formation of a niche for resident or therapeutic cell homing, in accordance with the nascent concept of matrix/cell therapy. This concept is currently paving the way towards RGTA^®^-based matrix and (stem) cell therapy combinations [23, 24].

## RGTA^®^ Pre-clinical models

The therapeutic potential of RGTA^®^ has been the subject of intense research, and over the past 25 years, the efficacy of these heparan sulfate analogs in improving wound healing in numerous pathological contexts and tissues has been demonstrated (Table 1).

**Table 1:** The effects of RGTA^®^ treatment in pre-clinical models

Model	Effects of RGTA^®^ Treatment	References
*Skin*		
Ischemic wounds in a diabetic context	-Acceleration of wound healing, total closure.	Tong *et al*. [25]
Necrotic ulcers	-Acceleration of ulcer closure. -Normalization of collagen III/I levels. -Reduced inflammation.	Barbier-Chassefiere *et al*. [26]
Full-thickness excisional wounds	-Increased local vascular response. -Increased skin strength.	Tong *et al*. [19]
Deep second degree burns	-Accelerated epidermal repair. -Decreased zone of stasis and extent of dermal remodeling.	Zakine *et al*. [27]
Burn-related skin fibrosis	-Normalization of collagen III/I levels. -Increased activity of MMP-2 and MMP-9.	Garcia-Filipe [28]
* Cornea*		
Corneal alkali burn	-Enhanced re-epithelialization. -Reduction in ocular inflammation. -Histological changes (reduced edema, fibrosis, blood vessels, inflammatory cells).	Brignole-Baudouin *et al*. [29]
Corneal alkali burn	-Decreased corneal inflammation. -Enhanced re-epithelialization. -Decreased neovascularization. -Healing of corneal ulcers following application 1 month post-injury.	Cejkova *et al*. [30]
Post-surgical corneal ulcers	-Increased corneal transparency. -Increased corneal thickness and epithelial cell organization. -Decreased myofibroblasts. -Increased nerve terminal density.	Riestra [31]
*Oral and Digestive Tract*		
5-fluorouracil induced mucositis	-Prevention of mucositis and reduction of area and thickness of lesions. -Protection of basement membrane.	Morvan *et al*. [32]
Irradiation induced oral mucositis	-Protection of lip mucosa from irradiation induced damage. -Improved tissue structure and mucosal thickness. -Reduced collagen deposition. -Decreased infiltration of leukocytes.	Mangoni *et al*. [21]
Periodontitis	-Reduced gingival tissue inflammation. -Restored alveolar bone.	Escartin *et al*. [33]
Periodontitis	-Restoration of gingival ECM. -Restoration of basement membrane. -Restoration of alveolar bone and interradicular bone.	Lallam-Laroye *et al*. [34]
Periodontitis	-Restoration of alveolar bone. -Restoration of periodontal attachment.	Lallam-Laroye *et al*. [35]
Ethanol-induced gastric lesion and acetic acid induced colitis	-Decreased severity of lesions.	Meddahi *et al*. [36]
Colonic anastomosis	-Increased resistance of anastomosis to leakage.	Meddahi *et al*. [37]
*Bone*, *Joint and Tendon*		
Craniotomy defects	-Increased bone filling rate. -Increased number of small vascular channels in newly formed bone. -Increased collagen I, III and fibronectin. -Increased osteoprogenitors.	Lafont *et al*. [38]
Partial thickness calvarial defects	-Accelerated bone healing. -Evidence of osteoblastic activity and bone remodeling. -No foreign body reaction observed, in contrast to controls.	Albo *et al*. [39]
Long bone defects	-Formation of new bone exhibiting continuity of the diaphysis, corticalization and a medullar canal.	Blanquaert [40]
Superficial digital flexor tendonitis (field study)	-Higher earnings post-injury compared to matched controls. -Earlier return to racing compared to matched controls.	Coudry *et al*. [41]
Superficial digital flexor tendonitis (controlled clinical trial)	-Decreased cross sectional area over time. -Higher number of races and victories. -Lower rate of reinjury.	Jacquet-Guibon, unpublished
*Muscle*		
Myocardial infarction (pig)	-Reduced loss of myocardial function. -Reduced infarct size. -Fecreased fibrotic tissue. -Preservation of myocytes. -Increased number of blood vessels.	Yamauchi *et al*. [42]
Myocardial infarction (baboon)	-Trend towards increased myocardial function.	Mullangi *et al*. [23]
Ischemic and denervated EDL muscle	-Inhibition of epimysial post-inflammatory reaction. -Increased peripheral zone and surviving muscle fibers. -Enhanced regeneration of muscle fibers and revascularization.	Desgranges *et al*. [43]
Ischemic EDL muscle	-Inhibition of ischemic process. -Acceleration of muscle fiber maturation. -Stimulation of muscle fiber regeneration. -Increased microvessel density.	Chevalier *et al*. [44]
EDL muscle crush	-Increased number of muscle fibers.	Gautron *et al*. [45]
EDL and soleus muscle crush and nerve cut	-Increased axonal growth and synaptic differentiation.	Aamiri *et al*. [46]
*Other Models*		
Sciatic nerve crush	-Decreased adherence of nerves to surrounding tissue.	Zuijdendorp *et al*. [47]
Implantation of foreign materials	-Improved foreign body reaction to implanted polyethylene terephthalate (PET). -RGTA^®^ coating of PET decreased the number of inflammatory giant cells. -Injected RGTA^®^ increased vascularization in tissue surrounding the implant and prevents deposition of collagen.	van Bilsen *et al*. [48]
Tympanic perforation repair	-Reduced thickness of the tympanic membrane. -Improved cellular reorganization.	Hellstrom, unpublished
Tauopathies	-Blocked neuronal uptake of tau fibrils.	Holmes *et al*. [49]

### Skin

The ability of RGTA^®^ to accelerate and improve the quality of skin wound healing is well documented in pre-clinical models, and the results of these studies have been instrumental for the transition of RGTA^®^ from the bench into the clinic. Indeed, RGTA^®^ has been successfully used to treat diabetic foot ulcers and chronic ulcers from other origins including prolonged pressure on the skin, misfunctioning of venous valves, due to sickle cell disease, following extensive burns and in cancer patients post-irradiation (see below).

In the context of treating diabetic ulcers, pre-clinical animal models, recapitulating lesions observed in human patients, are particularly relevant, since impaired wound healing may be attributed to several factors underlying the pathophysiology of this disease. For example, the production of advanced glycation end products that induce production of inflammatory molecules and affect collagen synthesis [50], and ischemia resulting from macro or microvascular disease [51]. In a model of ischemic wounds in diabetic rats, weekly intramuscular injection of RGTA^®^ enhanced the speed and total closure of wounds and improved the mechanical strength of scar tissue. This was associated with reduced inflammation, enhanced angiogenesis and increased collagen content (increased collagen I and decreased collagen III biosynthesis) [25]. Similarly, in a rat necrotic skin ulcer model, which more closely mirrors chronic wounds in humans compared to healthy skin repair models, topical administration of RGTA^®^ significantly accelerated ulcer healing and this was accompanied by decreased recruitment of leukocytes and normalization of collagen III/I ratios and improved dermal reconstruction [26]. Increased skin strength and vascular response following RGTA^®^ treatment were also observed in a rat excisional wound model [19].

Hypertrophic scar formation following burns and trauma remains a serious clinical problem and may be associated with a loss of function as well as disfigurement, significantly affecting patient quality of life. That RGTA^®^ improves wound healing quality and reduces the extent of fibrosis therefore has advantages not only from a functional point of view, but also from an aesthetic perspective. This extends the use of RGTA^®^ into reconstructive surgical procedures (see below) and in the case of burns. In a rat model of deep second degree burns, RGTA^®^ accelerated epidermal repair between days 3–7 post-treatment, as evidenced by the increased number of keratinocyte layers and staining for cytokeratin 14 [27]. Additionally, the size of the zone of stasis, which corresponds to delayed vascular lesions between the burn injury and adjacent healthy tissue, was smaller in RGTA^®^ treated animals. In a similar burn model, RGTA^®^ was capable of normalizing the ratio of collagen III/collagen I, which is often skewed towards high levels of collagen III in injured or in fibrotic tissue, and also increased the activity of MMP-2 and MMP-9, key regulators of ECM remodeling [28].

### Cornea

Significant potential exists for RGTA^®^ in the treatment of ocular surface diseases, with key studies showing that RGTA^®^ controls ocular surface inflammation and enhances corneal healing. In a rabbit corneal alkali burn model, a single treatment with RGTA^®^, at the time of injury, improved corneal healing, as assessed by reduced inflammation, enhanced re-epithelialization and specific histological patterns (decreased edema, fibrosis, blood vessels and inflammatory cells), with tissue organization closely mirroring that of normal cornea [29]. Later studies using a similar corneal alkali burn model, showed that RGTA^®^, applied 1 h following injury, accelerated corneal healing, associated with a decrease in corneal thickness and suppressed inflammation and neovascularization compared to controls. Importantly, RGTA^®^ also healed ulcers when applied 1 month after injury, indicating that RGTA^®^ not only exerts a post-injury protective effect, but also acts to actively promote healing of injured tissues [30]. Following corneal injury, levels of antioxidants are decreased in the cornea, creating an imbalance between prooxidants and antioxidants, and the resulting toxic oxygen and nitrogen products also contribute to corneal damage. In this model, RGTA^®^ reduced the expression of enzymes generating superoxide radicals and nitric oxide as well as proteolytic enzymes, thereby fulfilling a dual role: protecting the cornea from damage induced by excessive proteolysis as well as toxic oxygen and nitrogen products [30]. Furthermore, in a mouse model of corneal ulcers following photorefractive keratectomy (PRK), RGTA^®^ treatment increased the degree of corneal transparency and improved corneal epithelial cytoarchitecture and nerve regeneration, and decreased or delayed the presence of stromal myofibroblasts [52].

### Oral and digestive tract

Pre-clinical data demonstrating the efficacy of RGTA^®^ in oral and gastrointestinal tract associated lesions have also shown promise in the treatment of acute or chronic ulcers, during cancer treatment or following surgical procedures such as surgical anastomosis.

Oral mucositis, a complication of cancer treatment, particularly chemotherapy and radiation, causes atrophy of the mucosal lining of the mouth and the formation of ulcers, increasing the risk of infection and severely affecting patient quality of life. In a model of oral mucositis induced in hamsters by 5-fluorouracil (5-FU), RGTA^®^ prevented mucositis in 50 % of treated animals and significantly reduced the mean lesion volume in the remaining animals [32], effects that may be explained, at least in part, by the ability of RGTA^®^ to inhibit plasmin activity to normalize the balance of MMP-TIMPs, and to protect factors required for tissue healing such as TGFβ. The beneficial effects of RGTA^®^ in this model were also associated with protection of the basement membrane, evidenced by preservation of laminin and type IV collagen, consistent with the known protective role of RGTA^®^ on heparan binding matrix proteins. Importantly, treatment with RGTA^®^ did not interfere with the effects of 5-FU treatment on epithelial cell growth and no effects on body weight or blood cell counts were observed.

In a mouse irradiation model, RGTA^®^ also showed a strong anti-oxidative and protective effect resulting in reduced inflammation, less tissue destruction and improved recovery, highlighting its potential as a skin protector following radio-therapy [21].

RGTA^®^ also had marked effects in a hamster model of periodontitis, a common disease affecting the supporting tissue of the tooth, that can result in tooth loss or other associated health problems. Periodontitis is triggered by bacteria, and is characterized by inflammation and destruction of the gingival tissue and bone supporting the tooth. In this model, following the induction of disease, weekly intramuscular injections of RGTA^®^ reduced gingival tissue inflammation and bone loss, leading to the eventual restoration of alveolar bone and the regeneration of a functional periodontal ligament [33–35]. These effects were associated with an increase in the number of ERM (epithelial rests of Malassez) cells, which play roles in centenum repair and regeneration, and increased expression of bone morphogenic proteins (BMPs). Thus, RGTA^®^, *via* its ability to reestablish the cellular microenvironment and protect growth factor activity, is implicated in the survival, recruitment and proliferation of cells essential for regeneration.

In both ethanol-induced gastric lesion and acetic acid-induced colitis models, treatment with RGTA^®^ led to a marked reduction in the severity of lesions. In these models RGTA^®^ was administered before and shortly after induction of injury, and the protective effect of RGTA^®^ may be explained by its anti-inflammatory action and ability to protect the ECM and HBGFs required for tissue repair and angiogenesis [36].

Lastly, in a wound healing model of colonic anastomosis, RGTA^®^ increased the resistance of the anastomosis to leakage at early time points, highlighting its potential application as a post-surgical sealing agent [37]. This increased mechanical strength may at least in part be attributed to the ability of RGTA^®^ to inhibit the degradation and/or promote the synthesis of intestinal wall structural components such as collagen, possibly by increasing the bioavailability of TGFβ.

### Bone, joint and tendon

**Fig. 2:**
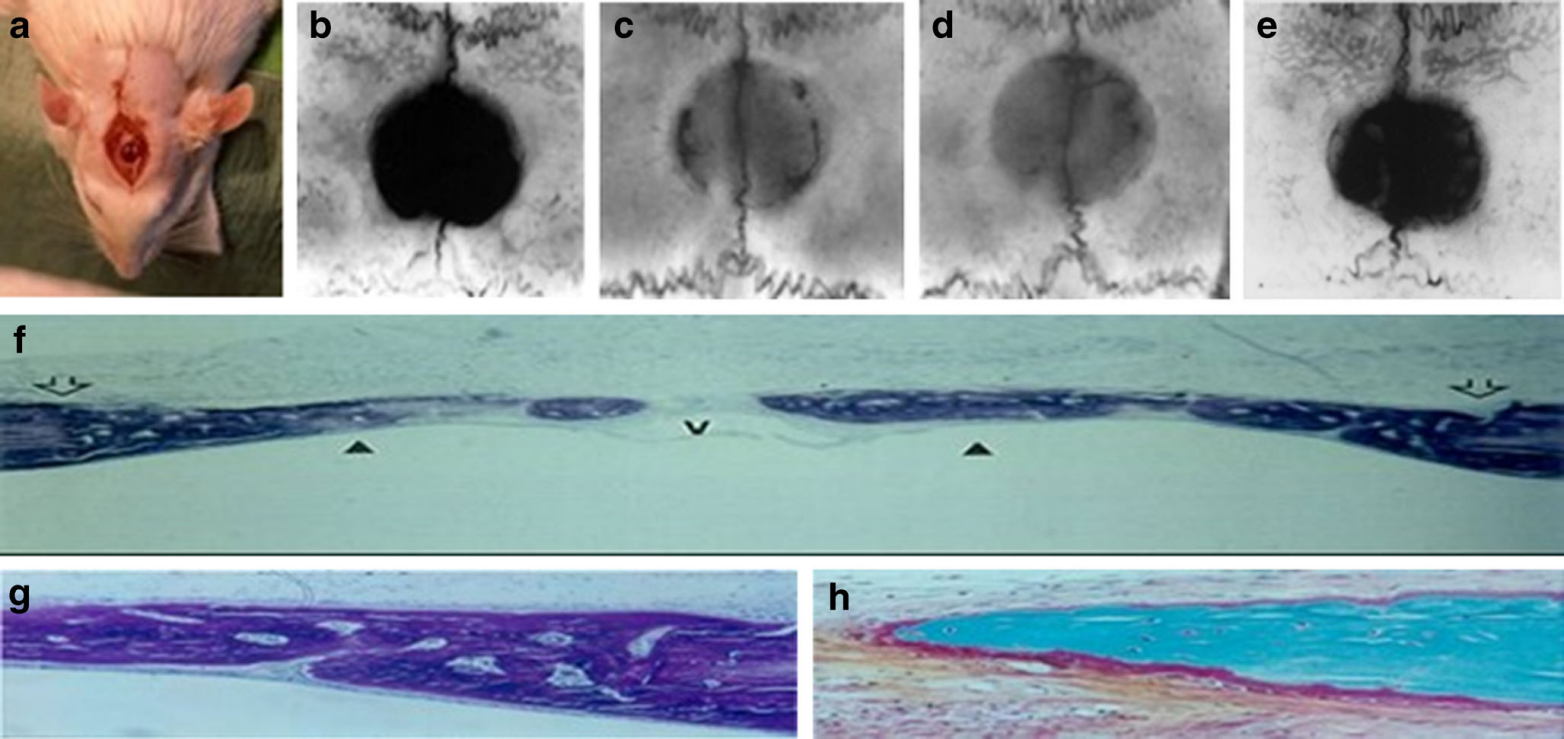
Filling of craniotomy defects. A. Operative view of the defect. X-ray of defects treated with a collagen plaster soaked in 30 ul of B. saline, C. RGTA^®^ solution (25 μg/ml) D. RGTA^®^ solution (100 μg/ml) and E. RGTA^®^ solution (400 ug/ml), after 4 weeks. F. Full macroscopic view of the skull defect with top arrows showing both sides of the defect and bottom arrows indicating new bone formation. Note the convex shape of the new bone structure, matching with expected original bone. v at the center indicates the saggital vein. G. Enlargement showing the lamellar organization of the flat bone as expected from a skull. H. alkaline phosphatase positive osteoblastic cells

As mentioned above, RGTA^®^ reduced bone loss in a hamster model of chronic periodontis, leading to the eventual restoration of alveolar bone and the regeneration of a functional periodontal ligament [33–35]. Some of the most remarkable effects of RGTA^®^ on tissue regeneration were also observed in bone using models of surgical defects and inflammatory bone destruction. In a rat model of craniotomy defects, where spontaneous healing does not occur, RGTA^®^ led to near complete bone filling, compared to controls where bone was only formed at the edges of the defect, and this was associated with an increase in osteoprogenitors and in type I and III collagen and fibronectin, suggesting that RGTA^®^ reactivated events paralleling those occurring during development [38, 53]. The new bone exhibited a higher number of small vascular channels where bone remodeling was observed [38]. Moreover, when the trephine hole was performed on both sides of the parietal bone including part of the sagittal suture, a newly formed suture was seen in the new bone in continuation with the remaining suture from the borders (Fig. 2). In support of these observations, RGTA^®^ also accelerated healing of bone-wounds in a model of partial thickness calvarial defect in rabbits [39]. In a rat long bone defect model, where a third of the total length of an adult femur was replaced by a pinned, demineralized, autologous bone soaked overnight in either saline or RGTA^®^, the RGTA^®^-impregnated bone prosthesis formed a new bone exhibiting corticalization and a medullar canal in 2 months. In contrast, non-organized bone cell colonization and formation of a callus was observed in control rats [24].

The effect of RGTA^®^ on tendon regeneration is best illustrated in the tendon to tendon slow and fast muscle crush models [45, 46] (see below). The efficacy of RGTA^®^ has also been demonstrated in the treatment of race horses with tendon rupture. In a field study involving 51 horses, comparison of a subset of RGTA^®^-treated horses with a matched control group, revealed that RGTA^®^-treated horses returned to racing earlier and performed better than non-treated horses [41]. These results were confirmed in a randomized, controlled trial of racing French Standardbred trotters presenting with superficial digital flexor tendonitis. Horses treated with a single, intra-lesion injection of RGTA^®^ exhibited improved healing, presented with a higher number of races and victories and benefited from a decreased reinjury rate compared to controls (Jacquet-Guibon, unpublished data). In the case of ligaments, the only reported effect of RGTA^®^ to date, is in the restoration of a functional periodontal ligament as mentioned above [35].

### Muscle

The potential of RGTA^®^ in the treatment of muscle injuries, including those induced by ischemia, is supported by several studies showing that RGTA^®^ can enhance muscle regeneration, reinnervation and revascularization.

In a model of acute myocardial infarction induced by ligation of the left circumflex artery in pigs, RGTA^®^ exerted a protective effect, evidenced by a 50 % reduction in the size of the infarct and higher recovery of the left ventricular ejection fraction compared to control treated animals. RGTA^®^-treated animals also exhibited reduced fibrotic tissue formation, increased numbers of blood vessels and preservation of myocytes, highlighting the potential for RGTA^®^ in the treatment of ischemic heart disease [42]. In support of these results, RGTA^®^ also improved the recovery of myocardial function following acute myocardial infarct (MI) in a Baboon model, with a reduced ejection fraction of 24.7 % observed in controls after 2 months compared to only 12 % in RGTA^®^-treated baboons [54].

Similarly, in a model of ischemic EDL (extensor digitorum longus) muscle in rats, a single injection of RGTA^®^ administered at the time of injury had marked effects at three levels. Firstly, the thickness of the epimysium of RGTA^®^ treated muscle was normal, comprising thin connective tissue, in comparison to the thick layer observed in controls. Secondly, RGTA^®^-treated muscle contained a larger peripheral zone containing a higher number of surviving muscle fibers and this area was interspersed with less connective tissue compared to controls and thirdly, the architecture and distribution of new fibers in the regenerating zone was increased in RGTA^®^-treated muscle, compared to controls, which exhibited few regenerated myotubes and necrotic muscle fibers. Additionally, vascularization was enhanced in RGTA^®^-treated muscle. Thus RGTA^®^ has a radial effect in muscle, providing both protection and regeneration of muscle fibers following injury [43, 44].

In a rat model of skeletal muscle damage induced by tendon to tendon crush, injured fast (EDL) and slow (soleus; SOL) muscle treated with a single injection of RGTA^®^, exhibited increased muscle fiber density and maturation, reduced fibrosis and more mature motor endplates and multi-axon connections [24, 45, 46]. Crushed tendon and overall muscle functional recovery were assessed by electromyography, and demonstrated that tendon to tendon crushed EDL muscles recovered total function (compared to non-injured contralateral muscle) *versus* 60 % in saline treated muscle. Similarly, SOL muscle recovered 85 % ± 15 % compared to no recovery in saline injected muscles [24].

### Other models

The efficacy of RGTA^®^-based matrix therapy has also been documented in several other models, including a rat model of sciatic nerve crush, where RGTA^®^ significantly reduced the force needed to break adhesions between the nerve and surrounding tissue [47], hence demonstrating anti-adhesion and anti-fibrosis properties.

The utility of RGTA^®^ as a substance to improve the foreign body reaction to implanted biomaterials such as poly(ethylene terephthalate)(PET) by increasing vascularization in the tissue surrounding the implant and reducing inflammation has also been demonstrated, highlighting its potential as a beneficial agent following implantation of medical devices [48].

In a rat model of tympanic membrane perforation, RGTA^®^ led to enhanced healing, reducing the thickness of the tympanic membrane to that of intact controls and improving cellular reorganization (Hellstrom *et al*., unpublished data). The potential of RGTA^®^ in the treatment of Alzheimer’s disease and other tauopathies has also been demonstrated, with a recent study showing that RGTA^®^ blocks neuronal uptake of tau fibrils *in vivo* [49].

### Modes of administration

RGTA^®^ can be administered both locally and systemically, and in certain preclinical models, one local application of RGTA^®^ was sufficient to regenerate injured tissues. In the rat EDL muscle crush model described above, systemically delivered RGTA^®^ was rapidly eliminated from the system, with specific uptake observed at the site of the injured tissue, supporting the model whereby RGTA^®^ binds heparan binding sites made available following destruction of HS in damaged tissue [10]. In this study, RGTA^®^ was detected in injured EDL muscle at least 1 week after injection, highlighting its stability and providing a rationale for its efficacy after a unique application. The pharmacokinetics and biodistribution of RGTA^®^ in other models of tissue damage are now being investigated and will be important for optimizing the dose and timing schedule of RGTA^®^.

## RGTA^®^ in the clinic

RGTA^®^ is already used in clinics in two commercially available products (OTR4120, alpha1-6 polycarboxylmethylsulfate glucose), one for the treatment of chronic skin lesions^1^ and the other for the treatment of corneal lesions.^2^ A second derivative, OTR4131 (alpha1-6 polycarboxylmethylsulfateacetate glucose), has shown efficacy in tendon and muscle regeneration and is now at the marketing registration stage.

### Skin wounds

RGTA^®^ matrix therapy has been successfully used to treat patients with a wide spectrum of wound types, since the mode of action is independent of the origin or cause of the lesion. Most cases concern chronic ulcers, associated with severe limb ischemia [55] (Roohi, in preparation), diabetes [56–58], burns [57, 59] pressure [57] and vascular defects [57], as well as in individual patients presenting with ulcers caused by sickle cell disease [60] radiation [61] and epidermolysis bullosa [62] (Table 2). In addition to chronic wounds, RGTA^®^ has also been used to treat patients with acute wounds [63] and following plastic skin surgery for scar reduction and orbital adhesion in centrofacial lifting [64]. To date, over 20 000 patients have already benefited from RGTA^®^ treatment, with no adverse effects yet reported.

**Table 2:** The effects of RGTA^®^ treatment in clinical cases or studies

Condition or Procedure	Effects of RGTA^®^ Treatment	References
*Skin*		
Non-healing ulcers due to severe limb ischemia	-Increased healing, increased granulation tissue. −50 % ulcers healed after 3 months.	Desgranges *et al*. [55]
Ischemia post-trauma	-Cases reports: rapid healing in finger and leg injuries.	Roohi (unpublished data); Desgranges *et al.* [55]
Sickle cell ulcer	-Case report: complete healing in a patient with a wound resistant to closure.	Hayek *et al*. [60]
Ulcers associated with Stewart-Bluefarb syndrome	-Case report: complete healing in a patient.	Hayek *et al*. [63]
Diabetic foot ulcers	-Complete wound closure in 60 % of patients and reduction in wound area in remaining patients.	Slim *et al*. [56]
Diabetic, pressure, vascular ulcers and burns	-Complete healing of 22 % of wounds at the end of the intervention period (up to 20 treatments sessions). -Reduction in wound size in remaining patients after 8 weeks. -Decreased pain.	Groah *et al*. [57]
Diabetic foot ulcers	-Complete healing of patients (12/12) exhibiting chronic resistance to previous therapy within 4–6 months.	Papanas *et al*. [58]
Heat burn	-Complete healing after 4 months.	Barritault *et al*. [59]
Abrasive and electric burns	-Case reports: healing and growth of granulation tissue.	Roohi (unpublished data)
Lower extremity ulcers associated with epidermolysis bullosa	-Case report: healing of chronic, non-healing ulcers within 4 weeks.	Malaq and Denis [62]
Traumatic and plastic surgery	-Case report: healing and growth of granulation tissue.	Roohi (unpublished data)
Plastic surgery: mammary reduction	-Reduced inflammation, itching and scarring post-surgery.	Zakine and Le Louarn [64]
Plastic surgery: centrofacial lift	-Reduced inflammation, swelling and bruising.	Zakine and Le Louarn [64]
Scalp lesions following surgery	-Complete closure of the wound after 12 weeks.	van Neck *et al*. [61]
*Cornea*		
Corneal neurotrophic ulcers	-Complete corneal healing in 72 % of patients after 8.7 weeks. -Decreased average ulcer area.	Aifa *et al*. [65]
Corneal neurotrophic ulcer	-Case report: corneal healing in a patient resistant to previous treatments.	Pison *et al*. [66]
Corneal healing post corneal collagen crosslinking	-Enhanced re-epithelialization compared to control treated eyes.	Kymionis *et al*. [67]
Herpes zoster corneal neurotrophic ulcer	-Case report: complete corneal healing after 2 weeks.	De Monchy *et al*. [68]
Persistant epithelial ulcers	−11 eyes treated, pain relief and improvement in 9 cases.	Chebbi *et al*. [69]
Limbal graft failure	-Successful limbal allografts after previous failures.	Maringe *et al*. [70]
Corneal perforation associated with Sjogren’s disease	-Corneal healing in all five treated patients.	Renault *et al*. [71]
Epithelial basement membrane dystrophy	-Corneal healing in five of six treated patients.	Labetoulle *et al*. [72]
Dry eye associated with corneal superficial punctate keratitis	-Reduced pain and ulcer size. -Improved visual acuity.	Gioganti-Aurégan (unpublished data)
Keratoconus	-Complete healing observed in 80 % of eyes (40 patients) compared to 15 % in the control group (*p* < 0.001). -Decreased ocular pain.	Gumus [73]
Corneal ulcer in a child with aniridia	-Corneal healing observed within 15 days.	Chiambaretta (unpublished data)

In a cohort of 12 patients presenting with non-healing ulcers due to severe limb ischemia (14 ulcers total), RGTA^®^ led to a significant decrease in the mean ulcer surface area compared to baseline at 4 weeks, and this was associated with increased granulation tissue [55]. After 3 months, 50 % of ulcers had completely healed and in a long term follow up of 9 patients, no reopening of closed ulcers was observed and no amputations were performed as a result of ulcers [55]. Similarly, in a study of diabetic patients presenting with non-healing foot ulcers, treatment with RGTA^®^ led to complete wound closure in 6 out of 10 patients, and a significant reduction in wound area in the remaining patients, after 10 weeks [56]. In a recent study involving type 2 diabetic patients with therapy resistant foot and lower extremity ulcers, local application of RGTA^®^ twice weekly led to complete healing in all patients (12/12) [58].

The remarkable response of patients to RGTA^®^ therapy in these clinical studies, particularly in light of the fact that many of these patients had failed to respond to standard treatment for a prolonged time, and were in therapeutic failure, despite provision of high-end care, underscores the potential for RGTA^®^ as a simple, first-line treatment in the management of chronic ulcers. Furthermore, in several studies, marked pain relief was observed by patients [57], and while the mechanism underlying this phenomenon remains unclear, this unique property may also contribute to the enhanced quality of life experienced by patients treated with RGTA^®^.

Accessibility to ECM proteins is essential for RGTA^®^ action, and indeed, maximum therapeutic efficacy was observed in cases where RGTA^®^ was applied following efficient removal of dead tissue from the wound. Given the inconvieniences associated with this procedure however (time consuming, painful, requiring significant expertise), future studies will be focused on novel treatment strategies aimed at bypassing the protective barrier and optimizing RGTA^®^ delivery to the wound site.

### Corneal ulcers

To date, RGTA^®^ has also been used to treat over 5000 patients with injuries and defects affecting the corneal epithelium and stroma, and its efficacy in the healing of this delicate tissue is supported by over 100 publications documenting clinical cases and studies (Table 2) [70–73]. Importantly, in these reports and in ongoing studies, RGTA^®^ was well tolerated, and no adverse side effects have been reported. In line with our observations in patients with chronic wounds, patients treated for corneal injuries also experienced decreased pain [69], which may reflect the anti-inflammatory properties of RGTA^®^.

Neurotrophic keratitis is a disease affecting the corneal epithelium that may have several underlying causes, including herpes keratitis, chemical or physical burns, topical drug toxicity, irradiation diabetes, specific ocular surgical procedures, among others. This degenerative disease is characterized by poor corneal healing which may progress to ulcers, infection and perforation as a result of chronic inflammation and cellular degradation or complete *de novo* vascularization. In a study of patients with severe corneal neurotrophic ulcers, treatment with RGTA^®^ (every 2 days) led to complete corneal healing in 8 out of 11 patients after approximately 9 weeks [65], supporting previous observations in individual patients presenting with neurotropic ulcers [66, 68]. In a second study involving 10 patients with severe corneal dystrophy or corneal ulcers, weekly treatment with RGTA^®^ over the course of 1 month led to improved healing and inflammation of most ulcers, and this was associated with reduced pain [69]. In this study, optimisation of the dose and number of treatments is needed however, since these effects were reversed in several patients following termination of treatment. It should be noted that in both studies, patients were resistant to previous treatments.

The benefit of RGTA^®^ as a healing agent following corneal surgery has also been demonstrated. In patients with keratoconus, whereby the corneal epithelium was removed using transepithelial phototherapeutic keratectomy, treatment with RGTA^®^ led to complete reepithelialization in 61.1 % of eyes after 3 days, compared to 11.1 % of control eyes [67].

Taken together these studies demonstrate that RGTA^®^ matrix based therapy is an efficacious and non-invasive approach to treat various injuries affecting the cornea and calls for future randomized studies with larger patient cohorts. The successful use of RGTA^®^ with other treatment approaches has also been demonstrated, for example bandage contact lenses which have been shown to be effective in reepithelialization, [74], or following treatment with crosslinking agents in keratoconus [73], highlighting its potential in combination treatment strategies.

## Discussion and future perspectives

Restoration of the microenvironment to facilitate natural tissue repair is an effective solution to treat diverse pathologies, as evidenced by the success of RGTA^®^-based matrix therapy in many pre-clinical models of tissue injury and in patients, with numerous other applications in the pre-clinical pipeline. RGTA^®^, with its simple and conservative mode of action, its glucose-based structure with only naturally existing substitutions (sulfates, carboxyl and acetate groups), its ability to specifically localize to sites of injury where it is retained throughout the restoration process [10], and its natural elimination as a matrix element with no evidence of toxicity [75], makes it a safe-profile product. Furthermore, RGTA^®^ is well tolerated in both pre-clinical models and in patients, with documented efficacy at very low doses. Several effects of RGTA^®^ obtained from animal models, including *the quality of tissue restoration* associated with (i) a reduction in fibrosis (ii) improvement of collagen I/III ratios (iii) microrevascularization in ischemic tissue, (iv) improved histological parameters and *functional recovery* associated with (i) increased skin breaking strength, (ii) reduced tissue adherence, (iii) improved recovery of heart, muscle and locomotion functions and (iv) corneal transparency, have also been observed humans following treatment with the first commercially available skin and corneal products. These benefits are also expected in upcoming clinical developments and provide the basis to test RGTA^®^ in other pathologies characterized by ECM destruction and inflammation. A remarkable and unexpected observation documented in several trials involving both corneal and skin injuries, was the pain relief experienced by patients shortly after RGTA^®^ treatment. This unexpected effect was not addressed in our initial animal studies, however understanding the mechanisms underlying this effect are of significant interest, since this is also an important factor affecting patient quality of life. Harnessing the full potential of RGTA^®^ in the clinic will require optimization of RGTA^®^ dosage (amount and frequency) and the mode of administration, in a tissue dependent manner and a thorough investigation of its efficacy in combination with other treatments, such as growth factors, cells and biomaterials.

## Footnotes

1 CACIPLIQ, see www.otr3.com list of trials, clinical and cases publication

2 CACICOL, see www.otr3.com list of trials, clinical and cases publication

## References

[1] Sarrazin S, Lamanna WC, Esko JD (2011). Heparan sulfate proteoglycans. Cold Spring Harb. Perspect. Biol..

[2] Hynes RO, Naba A (2012). Overview of the matrisome--an inventory of extracellular matrix constituents and functions. Cold Spring Harb. Perspect. Biol..

[3] Bonnans C, Chou J, Werb Z (2014). Remodelling the extracellular matrix in development and disease. Nat. Rev. Mol. Cell Biol..

[4] Cox TR, Erler JT (2011). Remodeling and homeostasis of the extracellular matrix: implications for fibrotic diseases and cancer. Dis. Model. Mech..

[5] Gallagher JT (2006). Multiprotein signalling complexes: regional assembly on heparan sulphate. Biochem. Soc. Trans..

[6] Eming SA, Krieg T, Davidson JM (2007). Inflammation in wound repair: molecular and cellular mechanisms. J. Invest. Dermatol..

[7] Papy-Garcia D, Barbier-Chassefière V, Rouet V, Kerros M-E, Klochendler C, Tournaire M-C (2005). Nondegradative sulfation of polysaccharides. Synthesis and structure characterization of biologically active heparan sulfate mimetics. Macromolecules.

[8] Jeanbat-Mimaud V, Barbaud C, Caruelle JP, Barritault D, Cammas-Marion S, Langlois V (2000). Bioactive functionalized polymer of malic acid for bone repair and muscle regeneration. J. Biomater. Sci. Polym. Ed..

[9] Ikeda Y, Charef S, Ouidja M-O, Barbier-Chassefière V, Sineriz F, Duchesnay A (2011). Synthesis and biological activities of a library of glycosaminoglycans mimetic oligosaccharides. Biomaterials.

[10] Meddahi A, Brée F, Papy-Garcia D, Gautron J, Barritault D, Caruelle J-P (2002). Pharmacological studies of RGTA(11), a heparan sulfate mimetic polymer, efficient on muscle regeneration. J. Biomed. Mater. Res..

[11] Barbier Chassefiere V. PhD Thesis: Synthese, Activites Pro-Cicatricielles et Etude Pharmacologique de l’OTR4120, RGTA Mimetique des Glycosaminoglycannes. Université Paris-Est Créteil (2008).

[12] Rouet V, Meddahi-Pellé A, Miao H-Q, Vlodavsky I, Caruelle J-P, Barritault D (2006). Heparin-like synthetic polymers, named RGTAs, mimic biological effects of heparin in vitro. J. Biomed. Mater. Res. A.

[13] Rouet V, Hamma-Kourbali Y, Petit E, Panagopoulou P, Katsoris P, Barritault D (2005). A synthetic glycosaminoglycan mimetic binds vascular endothelial growth factor and modulates angiogenesis. J. Biol. Chem..

[14] Mestries P, Alexakis C, Papy-Garcia D, Duchesnay A, Barritault D, Caruelle JP (2001). Specific RGTA increases collagen V expression by cultured aortic smooth muscle cells via activation and protection of transforming growth factor-beta1. Matrix Biol. J. Int. Soc. Matrix Biol..

[15] Albanese P, Caruelle D, Frescaline G, Delbé J, Petit-Cocault L, Huet E (2009). Glycosaminoglycan mimetics-induced mobilization of hematopoietic progenitors and stem cells into mouse peripheral blood: structure/function insights. Exp. Hematol..

[16] Tardieu M, Gamby C, Avramoglou T, Jozefonvicz J, Barritault D (1992). Derivatized dextrans mimic heparin as stabilizers, potentiators, and protectors of acidic or basic FGF. J. Cell. Physiol..

[17] Meddahi A, Lemdjabar H, Caruelle JP, Barritault D, Hornebeck W (1995). Inhibition by dextran derivatives of FGF-2 plasmin-mediated degradation. Biochimie.

[18] Meddahi A, Lemdjabar H, Caruelle JP, Barritault D, Hornebeck W (1996). FGF protection and inhibition of human neutrophil elastase by carboxymethyl benzylamide sulfonate dextran derivatives. Int. J. Biol. Macromol..

[19] Tong M, Zbinden MM, Hekking IJM, Vermeij M, Barritault D, van Neck JW (2008). RGTA OTR 4120, a heparan sulfate proteoglycan mimetic, increases wound breaking strength and vasodilatory capability in healing rat full-thickness excisional wounds. Wound Repair Regen. Off. Publ. Wound Heal. Soc. Eur. Tissue Repair Soc..

[20] Frescaline G, Bouderlique T, Huynh MB, Papy-Garcia D, Courty J, Albanese P (2012). Glycosaminoglycans mimetics potentiate the clonogenicity, proliferation, migration and differentiation properties of rat mesenchymal stem cells. Stem Cell Res..

[21] Mangoni M, Yue X, Morin C, Violot D, Frascogna V, Tao Y (2009). Differential effect triggered by a heparan mimetic of the RGTA family preventing oral mucositis without tumor protection. Int. J. Radiat. Oncol. Biol. Phys..

[22] Yue X-L, Lehri S, Li P, Barbier-Chassefière V, Petit E, Huang Q-F (2009). Insights on a new path of pre-mitochondrial apoptosis regulation by a glycosaminoglycan mimetic. Cell Death Differ..

[23] Mullangi C, Parhar R, Alshahid M, Kharabsheh S, Al Sugair A, Al Dayel F, *et al*.: New agent, Regenerating Agent (RGTA) and autologus bone marrow derived Cd34+ lineage stem cells transplantation for the treatment of acute myocardial infarction. Innovations. **200** (2006)

[24] Barritault D, Desgranges P, Meddahi-Pellé A, Denoix J, Saffar J (2016). RGTA-based matrix therapy - A New branch of regenerative medicine in locomotion. Joint Bone Spine.

[25] Tong M, Tuk B, Shang P, Hekking IM, Fijneman EMG, Guijt M (2012). Diabetes-impaired wound healing is improved by matrix therapy with heparan sulfate glycosaminoglycan mimetic OTR4120 in rats. Diabetes.

[26] Barbier-Chassefière V, Garcia-Filipe S, Yue XL, Kerros ME, Petit E, Kern P (2009). Matrix therapy in regenerative medicine, a new approach to chronic wound healing. J. Biomed. Mater. Res. A.

[27] Zakine G, Barbier V, Garcia-Filipe S, Luboinski J, Papy-Garcia D, Chachques JC (2011). Matrix therapy with RGTA OTR4120 improves healing time and quality in hairless rats with deep second-degree burns. Plast. Reconstr. Surg..

[28] Garcia-Filipe S, Barbier-Chassefiere V, Alexakis C, Huet E, Ledoux D, Kerros ME (2007). RGTA OTR4120, a heparan sulfate mimetic, is a possible long-term active agent to heal burned skin. J. Biomed. Mater. Res. A.

[29] Brignole-Baudouin F, Warnet JM, Barritault D, Baudouin C (2013). RGTA-based matrix therapy in severe experimental corneal lesions: safety and efficacy studies. J. Fr. Ophtalmol..

[30] Cejkova J, Olmiere C, Cejka C, Trosan P, Holan V (2014). The healing of alkali-injured cornea is stimulated by a novel matrix regenerating agent (RGTA, CACICOL20): a biopolymer mimicking heparan sulfates reducing proteolytic, oxidative and nitrosative damage. Histol. Histopathol..

[31] Riestra, A.C., Íñigo-Portugués, A., Artime, E., Braga, P., Merayo-Lloves, J., Alcalde, I.: Effectiveness of regenerative drops (CALCICOL) on corneal ulcers (post-PRK) in an experimental animal model. Invest. Ophthalmol. Vis. Sci. **55**(13), 5176 (2014)

[32] Morvan FO, Baroukh B, Ledoux D, Caruelle J-P, Barritault D, Godeau G (2004). An engineered biopolymer prevents mucositis induced by 5-fluorouracil in hamsters. Am. J. Pathol..

[33] Escartin Q, Lallam-Laroye C, Baroukh B, Morvan FO, Caruelle JP, Godeau G (2003). A new approach to treat tissue destruction in periodontitis with chemically modified dextran polymers. FASEB J. Off. Publ. Fed. Am. Soc. Exp. Biol..

[34] Lallam-Laroye C, Escartin Q, Zlowodzki A-S, Barritault D, Caruelle J-P, Baroukh B (2006). Periodontitis destructions are restored by synthetic glycosaminoglycan mimetic. J. Biomed. Mater. Res. A.

[35] Lallam-Laroye C, Baroukh B, Doucet P, Barritault D, Saffar J-L, Colombier M-L (2011). ReGeneraTing agents matrix therapy regenerates a functional root attachment in hamsters with periodontitis. Tissue Eng. Part A.

[36] Meddahi A, Alexakis C, Papy D, Caruelle J-P, Barritault D (2002). Heparin-like polymer improved healing of gastric and colic ulceration. J. Biomed. Mater. Res..

[37] Meddahi A, Benoit J, Ayoub N, Sézeur A, Barritault D (1996). Heparin-like polymers derived from dextran enhance colonic anastomosis resistance to leakage. J. Biomed. Mater. Res..

[38] Lafont J, Baroukh B, Berdal A, Colombier ML, Barritault D, Caruelle JP (1998). RGTA11, a new healing agent, triggers developmental events during healing of craniotomy defects in adult rats. Growth Factors Chur. Switz..

[39] Albo D, Long C, Jhala N, Atkinson B, Granick MS, Wang T (1996). Modulation of cranial bone healing with a heparin-like dextran derivative. J. Craniofac. Surg..

[40] Blanquaert F. Effets de molécules “Heparan-like” et de facteurs de croissance “Heparin-binding” sur des processus de réparation osseuse in vivo et sur des cellules ostéoblastiques in vitro. Université Paris-Est Créteil (1996)

[41] Coudry V, Dupays A, Carnicer D (2014). Long term follow-up of superficial digital flexor tendonitis treated by a single intralesional injection of a ReGeneraTing Agent (RGTA^®^) in 51 horses. J. Equine Vet. Sci..

[42] Yamauchi H, Desgranges P, Lecerf L, Papy-Garcia D, Tournaire MC, Moczar M (2000). New agents for the treatment of infarcted myocardium. FASEB J. Off. Publ. Fed. Am. Soc. Exp. Biol..

[43] Desgranges P, Barbaud C, Caruelle JP, Barritault D, Gautron J (1999). A substituted dextran enhances muscle fiber survival and regeneration in ischemic and denervated rat EDL muscle. FASEB J. Off. Publ. Fed. Am. Soc. Exp. Biol..

[44] Chevalier F, Arnaud D, Henault E, Guillevic O, Siñeriz F, Ponsen AC (2015). A fine structural modification of glycosaminoglycans is correlated with the progression of muscle regeneration after ischaemia: towards a matrix-based therapy?. Eur. Cell Mater..

[45] Gautron J, Kedzia C, Husmann I, Barritault D (1995). Acceleration of the regeneration of skeletal muscles in adult rats by dextran derivatives. C. R. Acad. Sci. III.

[46] Aamiri A, Mobarek A, Carpentier G, Barritault D, Gautron J (1995). Effects of substituted dextran on reinnervation of a skeletal muscle in adult rats during regeneration. C. R. Acad. Sci. III.

[47] Zuijdendorp HM, Smit X, Blok JH, Caruelle JP, Barritault D, Hovius SER (2008). Significant reduction in neural adhesions after administration of the regenerating agent OTR4120, a synthetic glycosaminoglycan mimetic, after peripheral nerve injury in rats. J. Neurosurg..

[48] van Bilsen P, Spronkmans C, Petersen A, Brouwer L, Huurdeman-Vincent J, Barritault D, *et al*. ReGeneraTing Agent (RGTA) improves the foreign body reaction against implanted poly(ethylene terephthalate) in rats. In: Effects of structure, morphology and heparin(−like) coatings on the tissue reaction to PET. p. 55–70 (2008)

[49] Holmes BB, DeVos SL, Kfoury N, Li M, Jacks R, Yanamandra K (2013). Heparan sulfate proteoglycans mediate internalization and propagation of specific proteopathic seeds. Proc. Natl. Acad. Sci. U. S. A..

[50] Hennessey PJ, Ford EG, Black CT, Andrassy RJ (1990). Wound collagenase activity correlates directly with collagen glycosylation in diabetic rats. J. Pediatr. Surg..

[51] LoGerfo FW, Coffman JD (1984). Current concepts. Vascular and microvascular disease of the foot in diabetes. Implications for foot care. N. Engl. J. Med..

[52] Alcalde I, Íñigo-Portugués A, Carreño N, Riestra AC, Merayo-Lloves JM (2015). Effects of new biomimetic regenerating agents on corneal wound healing in an experimental model of post-surgical corneal ulcers. Arch. Soc. Esp. Oftalmol..

[53] Lafont J, Baroukh B (1994). Derivatized dextrans (CMDBS) as promoters of bone healing. Factors influencing their effectiveness. Cells Mater..

[54] Mullangi C, Parhar R, Alshaid M, Kharabsheh S, Al Sugair A, Al Dayel F, *et al*. New Agent, Regenerating Agent (RGTA) And Autologus Bone Marr… : Innovations:Technology and Techniques in Cardiothoracic and Vascular Surgery [Internet]. LWW. [cité 31 mai 2016]. Disponible sur: http://journals.lww.com/innovjournal/Fulltext/2006/00140/New_Agent,_Regenerating_Agent__RGTA__And_Autologus.71.aspx

[55] Desgranges P, Louissaint T, Allaire E (2011). First clinical pilot study on critical ischemic leg ulcers with matrix therapy ReGeneraTing agent (RGTA^®^) technology. J. Wound Technol..

[56] Slim I, Tajouri H, Barritault D, Njah MK, Ach K, Chaieb MC (2012). Matrix protection therapy in diabetic foot ulcers: pilot study of CACIPLIQ20^®^. J. Wound Technol.

[57] Groah SL, Libin A, Spungen M, Nguyen K-L, Woods E, Nabili M (2011). Regenerating matrix-based therapy for chronic wound healing: a prospective within-subject pilot study. Int. Wound J..

[58] Papanas N, Demetzos C, Pippa N, Maltezos E, Tentolouris N (2016). Efficacy of a New heparan sulfate mimetic dressing in the healing of foot and lower extremity ulcerations in type 2 diabetes: a case series. Int. J. Low Extrem. Wounds.

[59] Barritault D, Al Harbi S, Filipe GS (2012). La thérapie matricielle une nouvelle branche de la médecine régénérative et ses applications dans le traitement des brulés: Du fondamental à la clinique. Brulure.

[60] Hayek S, Dibo S, Baroud J, Ibrahim A, Barritault D (2016). Refractory sickle cell leg ulcer: is heparan sulphate a new hope?. Int. Wound J..

[61] Van Neck JW, Zuidema YS, Smulders M, Balmus KJ (2011). Unexpected healing of radiation-induced scalp lesions with OTR4120, a heparan sulfate mimetic. Eur. J. Dermatol. EJD.

[62] Malaq AA, Denis B (2012). A rapid response to matrix therapy with RGTA in severe epidermolysis bullosa. Eplasty.

[63] Hayek S, Atiyeh B, Zgheib E (2015). Stewart-Bluefarb syndrome: review of the literature and case report of chronic ulcer treatment with heparan sulphate (Cacipliq20^®^). Int. Wound J..

[64] Zakine G, Le Louarn C (2010). First applications of matrix therapy in plastic and aesthetic surgery. Ann. Chir. Plast. Esthet..

[65] Aifa A, Gueudry J, Portmann A, Delcampe A, Muraine M (2012). Topical treatment with a new matrix therapy agent (RGTA) for the treatment of corneal neurotrophic ulcers. Invest. Ophthalmol. Vis. Sci..

[66] Pison A, Feumi C, Bourges J-L (2014). Healing of a resistant neurotrophic corneal ulcer using a new matrix therapy agent. J. Fr. Ophtalmol..

[67] Kymionis GD, Liakopoulos DA, Grentzelos MA, Tsoulnaras KI, Detorakis ET, Cochener B (2015). Effect of the regenerative agent poly(Carboxymethylglucose Sulfate) on corneal wound healing after corneal cross-linking for keratoconus. Cornea.

[68] De Monchy I, Labbé A, Pogorzalek N, Gendron G, M’Garrech M, Kaswin G (2012). Management of herpes zoster neurotrophic ulcer using a new matrix therapy agent (RGTA): a case report. J. Fr. Ophtalmol..

[69] Chebbi CK, Kichenin K, Amar N, Nourry H, Warnet JM, Barritault D (2008). Pilot study of a new matrix therapy agent (RGTA OTR4120) in treatment-resistant corneal ulcers and corneal dystrophy. J. Fr. Ophtalmol..

[70] Maringe E, Gueudry J, Aifa A, Delcampe A, Muraine M.: Topical treatment with a new matrix therapy agent (RGTA) in combination with limbal allograft in ocular surface desease and corneal anesthesia. Acta Ophthalmol (Copenh) [Internet]. (2013) [cité 21 juin 2016];91. Disponible sur: doi:10.1111/j.1755-3768.2013.S031.x

[71] Renault D, Lazreg S, Abdellah MB (2015). The use of matrix therapy in the treatment of corneal perforation. Invest. Ophthalmol. Vis. Sci..

[72] Labetoulle M, Rousseau A, M’Garrech M, Kaswin G, Dupas B, Baudouin C (2014). Efficacy of heparin sulfate mimetic polymer in Cogan’s epithelial dystrophy. Invest. Ophthalmol. Vis. Sci..

[73] Gumus, K., Gomas, G.M., Homem de Melo, M.S., Barritault, D., Karaküçük, S.: A new matrix therapy agent (RGTA) for faster corneal healing and less ocular discomfort following epi-off accelerated corneal crosslinking in progressive keratoconus. J. Refract. Surg. (2016) (in press) 10.3928/1081597X-20161206-07 28264130

[74] Kymionis GD, Liakopoulos DA, Grentzelos MA, Diakonis VF, Klados NE, Tsoulnaras KI (2014). Combined topical application of a regenerative agent with a bandage contact lens for the treatment of persistent epithelial defects. Cornea.

[75] Charef S, Tulliez M, Esmilaire L, Courty J, Papy-Garcia D (2010). Toxicological evaluation of RGTA OTR4120, a heparan sulfate mimetic. Food Chem. Toxicol. Int. J. Publ. Br. Ind. Biol. Res. Assoc..

